# Prevalence of Self-Managed Abortion Among Women of Reproductive Age in the United States

**DOI:** 10.1001/jamanetworkopen.2020.29245

**Published:** 2020-12-18

**Authors:** Lauren Ralph, Diana G. Foster, Sarah Raifman, M. Antonia Biggs, Goleen Samari, Ushma Upadhyay, Caitlin Gerdts, Daniel Grossman

**Affiliations:** 1 Advancing New Standards in Reproductive Health, Bixby Center for Global Reproductive Health, Department of Obstetrics, Gynecology and Reproductive Sciences, University of California, San Francisco; 2 Department of Population and Family Health, Mailman School of Public Health, Columbia University, New York, New York; 3 Ibis Reproductive Health, Oakland, California

## Abstract

**Importance:**

Increasing evidence indicates that people are attempting their own abortions outside the formal health care system. However, population-based estimates of experience with self-managed abortion (SMA) are lacking.

**Objective:**

To estimate the prevalence of SMA attempts among the general US population.

**Design, Setting, and Participants:**

This cross-sectional survey study was fielded August 2 to 17, 2017 among English- and Spanish- speaking, self-identified female panel members from the GfK web-based KnowledgePanel. Women ages 18 to 49 years were approached to complete a 1-time survey. Data were analyzed from September 22, 2017, to March 26, 2020.

**Main Outcomes and Measures:**

SMA was defined as “some women may do something on their own to try to end a pregnancy without medical assistance. For example, they may get information from the internet, a friend, or family member about pills, medicine, or herbs they can take on their own, or they may do something else to try to end the pregnancy.” SMA was assessed using the question, “Have you ever taken or used something on your own, without medical assistance, to try to end an unwanted pregnancy?” Participants reporting SMA were asked about methods used, reasons, and outcomes. Factors associated with SMA experience, including age, race/ethnicity, socioeconomic status, nativity, reproductive health history, and geography, were assessed. Projected lifetime SMA prevalence was estimated using discrete-time event history models, adjusting for abortion underreporting.

**Results:**

Among 14 151 participants invited to participate, 7022 women (49.6%) (mean [SE] age, 33.9 [9.0] years) agreed to participate. Among these, 57.4% (95% CI, 55.8%-59.0%) were non-Hispanic White, 20.2% (95% CI, 18.9%-21.5%) were Hispanic, and 13.3% (95% CI, 12.1%-14.5%) were non-Hispanic Black; and 15.1% (95% CI, 14.1%-16.3%) reported living at less than 100% federal poverty level (FPL). A total of 1.4% (95% CI, 1.0%-1.8%) of participants reported a history of attempting SMA while in the US. Projected lifetime prevalence of SMA adjusting for underreporting of abortion was 7.0% (95% CI, 5.5%-8.4%). In bivariable analyses, non-Hispanic Black (prevalence ratio [PR], 3.16; 95% CI, 1.48-6.75) and Hispanic women surveyed in English (PR, 3.74; 95% CI, 1.78-7.87) were more likely than non-Hispanic White women to have attempted SMA. Women living below 100% of the FPL were also more likely to have attempted SMA compared with those at 200% FPL or greater (PR, 3.43; 95% CI, 1.83-6.42). At most recent SMA attempt, 20.0% (95% CI, 10.9%-33.8%) of respondents used misoprostol, 29.2% (95% CI, 17.5%-44.5%) used another medication or drug, 38.4% (95% CI, 25.3%-53.4%) used herbs, and 19.8% (95% CI, 10.0%-35.5%) used physical methods. The most common reasons for SMA included that it seemed faster or easier (47.2% [95% CI, 33.0%-61.8%]) and the clinic was too expensive (25.2% [95% CI, 15.7%-37.7%]). Of all attempts, 27.8% (95% CI, 16.6%-42.7%) of respondents reported they were successful; the remainder reported they had subsequent facility-based abortions (33.6% [95% CI, 21.0%-49.0%]), continued the pregnancy (13.4% [95% CI, 7.4%-23.1%]), had a miscarriage (11.4% [95% CI, 4.2%-27.5%]), or were unsure (13.3% [95% CI, 6.8%-24.7%]). A total of 11.0% (95% CI, 5.5%-21.0%) of respondents reported a complication.

**Conclusions and Relevance:**

This cross-sectional study found that approximately 7% of US women reported having attempted SMA in their lifetime, commonly with ineffective methods. These findings suggest that surveys of SMA experience among patients at abortion clinics may capture only one-third of SMA attempts. People’s reasons for attempting SMA indicate that as abortion becomes more restricted, SMA may become more common.

## Key Points

**Question:**

What is the prevalence of self-managed abortion (SMA) among US women of reproductive age?

**Findings:**

In this cross-sectional survey of 7022 women aged 18 to 49, 1.4% reported ever having attempted SMA. Using age at SMA attempt and adjusting for underreporting of abortion, it is estimated that 7.0% of US women will attempt SMA at some point in their lives.

**Meaning:**

These findings suggest that SMA is occurring in the US, highlighting the need for innovative models to ensure people have access to the safest and most effective methods of SMA, particularly where facility-based care is inaccessible.

## Teaser

This cross-sectional study examines the prevalence of and sociodemographic characteristics associated with reporting attempted self-managed abortion among US women.

## Introduction

Self-managed abortion (SMA) is any action taken to end a pregnancy outside the formal health care system and includes self-sourcing the World Health Organization (WHO)–recommended medications (ie, mifepristone and misoprostol), ingesting herbs or other drugs, and physical methods, such as insertion of foreign objects into the uterus.^1^ People’s reasons for SMA are varied, and include both difficulty accessing facility-based abortion care and a preference for a more private or natural abortion experience.^2,3,4^

Recent evidence sheds some light on the practice of SMA in the United States, highlighting online searches for information about ways to end a pregnancy without clinical supervision,^5,6,7,8^ requests for medication abortion through an online telemedicine service,^2^ and preferences for SMA outside the health care system.^9^ Abortion clinics and practitioners report caring for an increasing number of individuals who have attempted SMA,^10,11^ and researchers speculate that the decline in the US of facility-based abortion care may, at least in part, be driven by increases in abortions occurring outside of the formal health care system not captured by current measurement efforts.^12^

Global estimates suggest that 17 million less safe abortions, including use of misoprostol without information or support from a trained individual and an additional 8 million unsafe abortions using other techniques occur annually, with 97% taking place in developing countries.^13^ Data on SMA in the US come primarily from studies of people accessing abortion and other primary or reproductive health care, which may miss the experiences of people unable to access facility-based care. In this research, between 2% and 13% of people accessing facility-based abortion and other health care reported having ever attempted to end a pregnancy on their own.^3,14,15,16^ In the only population-based study available, conducted among reproductive-aged women in Texas, 1.7% reported having ever self-managed an abortion.^17^ To date, there are no national, population-based estimates of the prevalence of SMA or people’s experience with SMA in the US, to our knowledge.

As access to legal, facility-based abortion care in the US becomes more difficult owing to increased restrictions on abortion provision, there is increasing interest in understanding and documenting SMA. In this study, we use nationally representative survey data to estimate the proportion of US women of reproductive age who have ever attempted SMA, hypothesizing that SMA experience will be more common among populations that lack access to health care or experience stigma associated with abortion; project lifetime prevalence of SMA; and among those with SMA experience, describe methods used, effectiveness of those methods, reasons for choosing SMA, and complications experienced.

## Methods

This cross-sectional study was prepared in accordance with the Strengthening the Reporting of Observational Studies in Epidemiology (STROBE) reporting guideline for reporting on cross-sectional studies. All study activities were approved by the University of California, San Francisco, institutional review board. Study participants provided electronic informed consent after reading about the study online through the GfK platform.

### Sample

Data for this analysis were collected by the GfK Group (now Ipsos) from members of its web-based KnowledgePanel. Panel members are recruited from a probability sample of US addresses to be representative of the noninstitutionalized US population; those who agree to join the panel are regularly invited to participate in online survey activities, and if needed are provided with the technology to do so.^18^ Between August 2 and 17, 2017, GfK invited 14 151 eligible panel members via email to complete a 53-item, cross-sectional survey designed by University of California, San Francisco, researchers on women’s experiences and opinions related to reproductive health care. Eligibility was defined as self-identifying as female and being aged 18 to 49 years. Reminders were sent 3 and 8 days after initial contact. Participants were reimbursed for participation through GfK’s points program, in which panelists receive cash-equivalent checks ranging from $4 to $6 per month depending on their participation.

## Measures

The primary outcome of interest was experience with SMA, assessed using the question, “Have you ever taken or used something on your own, without medical assistance, to try to end an unwanted pregnancy?” A longer description of SMA, developed using cognitive interviews,^19^ preceded this question: “As we mentioned earlier, some women may do something on their own to try to end a pregnancy without medical assistance. For example, they may get information from the internet, a friend, or family member about pills, medicine, or herbs they can take on their own, or they may do something else to try to end the pregnancy. Have you ever taken or used something on your own, without medical assistance, to try to end an unwanted pregnancy?”

Participants who responded affirmatively about SMA experience were asked a series of follow-up questions about their most recent attempt including what methods they used; in what year the attempt took place (from a drop-down menu); in what state they were living at the time (from a drop-down menu), with the option to write-in another country; whether the methods worked to end the pregnancy; whether they experienced any medical complications, defined as something requiring “treatment by a doctor or nurse”; and their reasons for “ending the pregnancy on their own instead of going to a clinic.” All questions were close-ended but included an other, something else, or not sure option. For methods, efficacy, and reasons, participants could select multiple response options and write-in additional responses. If they selected herbs for method type or yes to experiencing any complications, an open-ended follow-up question probed for additional detail. Using their self-reported age and the year of SMA attempt, we calculated their age at the time of SMA. We collapsed states into geographic regions defined by the US Census.^20^ Finally, we asked about past-year and lifetime experience with abortion and the number of financial and logistical barriers experienced when accessing reproductive health care in the past 3 years.

Sociodemographic information is routinely collected by GfK for all KnowledgePanel participants. In this analysis, we used panel variables on participant’s age in years, race/ethnicity, highest level of education completed, marital status, place of birth (US vs another country), parity, current state of residence, and past-year material hardship (defined as ability to come up with $2000).^21^ Using responses to household income and size questions, we calculated household percentage of the federal poverty level (FPL) using 2017 Census thresholds.^22^ For most analyses, we used a composite variable combining race/ethnicity with language of the survey (English vs Spanish).

### Statistical Analysis

We used descriptive statistics to describe participant characteristics, generate estimates of the proportion who had ever attempted SMA, and describe experiences with SMA. For all SMA outcomes, we removed attempts that occurred outside the US and those that used emergency contraception (EC) before confirming the pregnancy as their only method of SMA, since this may have represented appropriate use of EC and not abortion.

We estimated the weighted proportion with SMA experience within subgroups of age, race/ethnicity, and other sociodemographic characteristics and described the bivariable association between these characteristics and experience with SMA using Poisson regression. Exponentiated coefficients represent prevalence ratios (PRs).

We generated a multivariable Poisson regression model estimating factors associated with SMA. To establish the temporal association between covariates and SMA experience, we only included covariates that are time-invariant (race/ethnicity), preceded the attempted SMA (completion of high school), or serve as a proxy for a time-invariant construct (survey language). We originally planned to use place of birth in the multivariable analysis; however, there was higher nonresponse to this question than language of the survey (5% vs 0%). Given that some participants reported SMA prior to age 18 years, we conducted a second, sensitivity analysis with the subset of participants who reported that their SMA attempt occurred at age 18 or older.

From our cross-sectional data that include people ages 18 to 49 year, we projected lifetime experience with SMA using discrete-time event history models. Using participants’ reports of their age at the time of most recent SMA attempt and their current age, we applied the tfr2 command in Stata version 15.1 (StataCorp) to estimate 5-year age-specific rates of SMA and then, based on these age-specific rates, sum across the age groups to generate an estimate of lifetime prevalence.^23,24^ For all analyses, we applied survey weights using the svyset command in Stata and weights provided by GfK designed to reweight the sample such that it is representative of noninstitutionalized women ages 18 to 49 years living in the US with respect to age, race/ethnicity, census region, education, household income, and language. To ensure the weighting resulted in a sample representative of our target population of women of reproductive age, we descriptively compared our study sample with the 2015 to 2017 National Survey of Family Growth (NSFG), using public use data files.^25^

Given that underreporting of abortion is a common challenge in survey research,^26,27,28^ we assumed that respondents underreported SMA. We adjust for underreporting by comparing reported past-year abortions in the GfK to estimates derived from surveys of abortion facilities, which are generally considered more complete.

## Results

Among 14 151 eligible women invited to participate, a total of 7022 women (mean [SE] age, 33.9 [9.0] years) completed the survey; 57.4% (95% CI, 55.8%-59.0%) of participants were non-Hispanic White, 20.2% (95% CI, 18.9%-21.5%) were Hispanic, and 13.3% (95% CI, 12.1%-14.5%) were non-Hispanic Black. A total of 15.1% (95% CI, 14.1%-16.3%) of respondents were living below 100% FPL (Table 1). Compared with the 2015 to 2017 NSFG, the sample is comparable to women of reproductive age in the US with respect to age, race/ethnicity, and high school completion. However, the GfK sample underrepresents people living below 100% FPL compared with the 2015-2017 NSFG (15.2% vs 22.0%) (eTable in the Supplement).

**Table 1.** Demographic and Reproductive Health History of the Study Sample

Characteristic	Unweighted No. (N = 7022)	Weighted % (95% CI)^a^
Age, y		
18-24	536	19.4 (17.8-21.1)
25-29	1098	16.8 (15.6-18.0)
30-39	2697	32.4 (30.1-33.8)
40-49	2691	31.4 (30.1-32.8)
Race/ethnicity		
Non-Hispanic White	4441	57.4 (55.8-59.0)
Non-Hispanic Black	680	13.3 (12.1-14.5)
Hispanic	1411	20.2 (18.9-21.5)
Non-Hispanic Asian or Pacific Islander	247	6.7 (5.8-7.8)
Non-Hispanic multiracial or other race/ethnicities	243	2.4 (2.0-2.9)
Income, % Federal poverty level		
<100	1274	15.1 (14.1-16.3)
100%-199	1248	16.0 (15.0-17.2)
≥200	4500	68.8 (67.4-70.3)
Material hardship, confidence in ability to come up with $2000		
Not at all or only slightly confident	2993	42.4 (40.8-44.0)
Somewhat or very confident	3239	46.8 (45.2-48.4)
Not asked or refused	790	10.9 (9.9-11.9)
Educational attainment		
<High school	288	9.9 (8.8-11.2)
High school diploma	1033	22.6 (21.1-24.1)
Some college	2314	32.4 (30.1-33.9)
≥Bachelor’s degree	3387	35.1 (33.7-36.6)
Marital status		
Married	3898	53.3 (51.7-54.9)
Widowed, divorced, or separated	783	8.2 (7.5-9.0)
Never married	1650	28.4 (26.8-30.0)
Living with partner	691	10.1 (9.2-11.1)
Language of completed survey		
English	6420	91.1 (90.2-92.0)
Spanish	602	9 (8.0-9.8)
Place of birth		
US	5672	79.3 (78.0-80.6)
Outside the US	946	15.9 (14.7-17.2)
Missing or refused	404	4.8 (4.3-5.5)
Parity		
0	2639	41.4 (39.7-43.0)
1	1243	16.2 (15.1-17.3)
2	1712	22.7 (21.5-24.0)
3	886	12.2 (11.3-13.3)
≥4	510	7.0 (6.3-7.8)
Refused	32	4.9 (3.1-7.8)
Current geographic region of residence		
Northeast (Connecticut, Maine, Massachusetts, New Hampshire, and Rhode Island)	278	4.1 (3.5-4.8)
Mid-Atlantic (New Jersey, New York, and Pennsylvania)	867	12.7 (11.6-13.8)
East North Central (Illinois, Indiana, Michigan, Ohio, and Wisconsin)	1150	13.9 (12.9-15.0)
West North Central (Iowa, Kansas, Minnesota, Missouri, Nebraska, North Dakota, and South Dakota)	577	6.5 (5.8-7.3)
South Atlantic (Delaware, Florida, Georgia, Maryland, North Carolina, South Carolina, Virginia, District of Columbia, and West Virginia)	1333	20.5 (19.2-21.9)
East South Central (Alabama, Kentucky, Mississippi, and Tennessee)	330	5.1 (4.4-5.9)
West South Central (Arkansas, Louisiana, Oklahoma, and Texas)	804	12.5 (11.5-13.6)
Mountain (Arizona, Colorado, Idaho, Montana, Nevada, New Mexico, Utah, and Wyoming)	523	6.7 (6.0-7.5)
Pacific (Alaska, California, Hawaii, Oregon, and Washington)	1160	17.8 (16.6-19.2)
Lifetime abortion		
No	5936	86 (84.4-86.5)
Yes	1011	13 (12.4-14.4)
Refused	75	1 (0.8-1.6)
Past-year abortion		
No	6893	98.0 (97.4-98.4)
Yes	54	0.6 (0.6-1.2)
Refused	75	0.8 (0.9-1.6)
No. of barriers to reproductive health care in past 3 y		
None or never tried	4331	64.8 (63.3-66.3)
1	1027	13.0 (12.0-14.0)
2	590	7.8 (7.0-8.6)
≥3	1030	13.7 (12.6-14.8)
Refused	44	0.8 (0.5-1.1)

^a^ Values are column percentages. Percentages are weighted so that responses are representative of noninstitutionalized women ages 18 to 49 years living in the US with respect to age, race/ethnicity, region of residence, education, household income, and language.

The proportion of the study sample who reported ever having attempted SMA while living in the US was 1.4% (95% CI, 1.0%-1.8%). Few respondents (<1%) skipped the SMA question (eFigure 1 in the Supplement). Adjusted for estimated underreporting of facility-based abortions, SMA prevalence was 2.0% (95% CI, 1.5%-2.6%), Table 2. Specifically, 1.00% of women ages 18 to 45 years in our sample reported a past-year abortion, which is lower than national estimates of 1.45%.^10^ We therefore multiplied our overall estimates of SMA by a factor of 1.45 in order to account for underreporting of abortion.

**Table 2.** Estimates of SMA Attempts, Overall and Adjusted for Underreporting of Abortion

Outcome	Unweighted No./Sample size	Weighted % (95% CI)^a^
History of SMA		
Overall	106/6953	1.51 (1.16-1.98)
While living in the US	92/6938	1.36 (1.02-1.81)
Adjusted for underreporting	NA	1.97 (1.48-2.62)
Estimated lifetime prevalence of SMA		
Overall	NA	4.82 (3.85-5.78)
Adjusted for underreporting	NA	6.99 (5.58-8.38)

Abbreviation: SMA, self-managed abortion.

^a^ Values are row percentages. All estimates exclude participants who reported using emergency contraception before confirming pregnancy as their only method of SMA.

Using discrete-time event models, women’s projected lifetime prevalence of SMA was 4.8% (95% CI, 3.8%-5.8%) (Table 2). Estimated age-specific rates indicated that experience with SMA peaks in young adulthood and then declines with age (eFigure 2 in the Supplement). Adjusted for underreporting, projected lifetime prevalence of SMA was estimated at 7.0% (95% CI, 5.5%-8.4%), (Table 2).

In bivariable analysis, non-Hispanic Black women (PR, 3.16, 95%, 1.48-6.75) and Hispanic women completing the survey in English (PR, 3.74; 95% CI, 1.78-7.87) were more likely than non-Hispanic White women to have attempted SMA in the US. Women born outside the US were more likely than those born in the US to have ever attempted SMA (PR, 2.12; 95% CI, 1.07-4.20). Women currently living below 100% of FPL were also more likely to have ever attempted SMA (PR, 3.43; 95% CI, 1.83-6.42) compared with women living at or above 200% FPL. Women who reported 2 barriers to reproductive health care were 2-fold more likely to report SMA experience compared with those who reported no barriers (PR, 2.38; 95% CI, 1.04-5.42). Finally, participants born outside the US were more likely to have ever attempted SMA in the US compared with those born in the US (PR, 2.12; 95% CI, 1.07-4.20) (Table 3).

**Table 3.** Bivariable Associations Between Experience Attempted SMA and Sociodemographic Characteristics

Characteristic	Unweighted No./Sample size	Weighted % (95% CI)^a^	PR (95% CI)
Age, y			
18-24	6/529	1.3 (0.5-3.0)	1 [Reference]
25-29	20/1087	1.8 (1.0-3.3)	1.40 (0.49-4.05)
30-39	47/2667	1.6 (1.07-2.5)	1.27 (0.49-3.32)
40-49	33/2655	1.0 (0.6-1.6)	0.74 (0.27-2.04)
Race/ethnicity			
Non-Hispanic white	42/4396	0.8 (0.5-1.3)	1 [Reference]
Non-Hispanic Black	16/671	2.6 (1.4-4.8)	3.16 (1.48-6.75)^a^
Hispanic: survey in English	20/797	3.1 (1.7-5.6)	3.74 (1.78-7.87)^a^
Hispanic: survey in Spanish	22/588	1.6 (0.6-3.8)	1.90 (0.71-5.11)
Non-Hispanic Asian or Pacific Islander	1/244	0.4 (0.1-2.7)	0.45 (0.06-3.38)
Non-Hispanic multiracial or other	5/242	1.7 (0.6-4.8)	2.04 (0.65-6.41)
Income, % FPL			
<100	37/1248	3.4 (2.1-5.3)	3.43 (1.83-6.42)^b^
100-199	25/1232	1.2 (0.6-2.4)	1.22 (0.55-2.71)
≥200	44/4458	1.0 (0.6-1.5)	1 [Reference]
Material hardship: confidence in ability to come up with $2000			
Not at all or only slightly confident	59/2965	1.8 (1.2-2.6)	1.54 (0.84-2.84)
Somewhat or very confident	39/3209	1.2 (0.7-1.9)	1 [Reference]
Not asked or refused	8/764	0.5 (0.2-1.2)	0.46 (0.18-1.16)
Highest level of educational attainment			
<High school diploma	10/283	3.1 (1.6-6.2)	1 [Reference]
High school diploma	10/1018	0.8 (0.4-2.0)	0.57 (0.09-0.83)
Some college	43/2287	1.5 (1.1-2.35)	0.50 (0.23-1.09)
≥Bachelor’s degree	43/3350	1.1 (0.6-1.9)	0.35 (0.14-0.84)^c^
Marital status			
Married	45/3857	1.1 (0.7-1.8)	1 [Reference]
Widow, divorced, or separated	19/768	2.5 (1.2-5.1)	2.19 (0.91-5.21)
Never married	26/1635	1.2 (0.7-2.1)	1.09 (0.53-2.24)
Living with partner	16/678	2.3 (1.3-4.3)	2.09 (0.97-4.51)
Place of birth			
Outside the US	32/922	2.5 (1.4-4.5)	2.12 (1.07-4.20)^c^
US	69/5628	1.2 (0.9-1.6)	1 [Reference]
Missing or refused	5/388	0.9 (0.3-2.4)	0.35 (0.11-1.12)
Parity			
0	28/2614	1.2 (0.7-2.1)	1 [Reference]
1	19/1230	1.1 (0.5-2.3)	0.86 (0.34-2.19)
≥2	58/3080	1.6 (1.1-2.3)	1.30 (0.68-2.46)
Current geographic region			
Northeast (Connecticut, Maine, Massachusetts, New Hampshire, and Rhode Island)	3/268	0.5 (1.0-2.4)	1.17 (0.12-10.53)
Mid-Atlantic (New Jersey, New York, and Pennsylvania)	4/863	0.4 (0.1-2.0)	1 [Reference]
East North Central (Illinois, Indiana, Michigan, Ohio, and Wisconsin)	19/1137	1.4 (0.7-2.6)	3.17 (0.59-16.98)
West North Central (Iowa, Kansas, Minnesota, Missouri, Nebraska, North Dakota, and South Dakota)	6/574	0.9 (0.3-2.5)	2.09 (0.33-13.33)
South Atlantic (Delaware, Florida, Georgia, Maryland, North Carolina, South Carolina, Virginia, District of Columbia, and West Virginia)	17/1316	0.9 (0.4-2.0)	2.02 (0.35-11.77)
East South Central (Alabama, Kentucky, Mississippi, and Tennessee)	2/326	1.0 (0.2-4.2)	2.32 (0.28-19.41)
West South Central (Arkansas, Louisiana, Oklahoma, and Texas)	18/793	2.4 (1.2-4.5)	5.53 (1.04-29.52)^c^
Mountain (Arizona, Colorado, Idaho, Montana, Nevada, New Mexico, Utah, and Wyoming)	7/516	1.2 (3.4-4.5)	2.90 (0.39-21.71)
Pacific (Alaska, California, Hawaii, Oregon, and Washington)	30/1145	2.5 (1.5-4.1)	5.82 (1.14-29.62)^c^
Abortion history			
No	48/5907	0.7 (0.5-1.0)	1 [Reference]
Yes	56/998	5.7 (3.8-8.4)	8.16 (4.64-14.36)^b^
Refused	2/33	4.4 (0.9-20.0)	6.41 (1.32-30.96)^c^
Barriers to reproductive health care in past 3 y, No.			
None or never tried to access	31/4293	0.7 (0.4-1.3)	1 [Reference]
1	12/1019	1.3 (0.5-2.9)	1.75 (0.63-4.85)
2	18/584	1.7 (0.9-3.1)	2.38 (1.04-5.42)^c^
≥3	45/1015	4.5 (3.0-6.7)	6.32 (3.18-12.49)^b^
Missing or refused	0/27	0	NA

Abbreviations: FPL, federal poverty level; NA, not applicable; PR, Prevalence Ratio; SMA, self-managed abortion.

^a^ *P* < .01.

^b^ *P* < .001.

^c^ *P* < .05.

In multivariable analysis restricted to covariates that we could establish preceded the SMA attempt, non-Hispanic Black women (adjusted PR [aPR], 3.09; 95% CI, 1.36-7.02) and Hispanic women completing the survey in English (aPR, 3.60; 95% CI, 1.59-8.14) were more likely than non-Hispanic White women to report ever attempting SMA. Women with less than a high school education were also more likely to have ever attempted SMA (PR, 2.99; 95% CI, 1.37-6.53) compared with women with a high school diploma or above (Table 4).

**Table 4.** Multivariable Poisson Regression Models of Reporting Ever Attempting SMA in the United States

Characteristic	Adjusted SMA PR (95% CI)	
	Full sample (n = 6953)^a^	Excluding participants whose SMA attempt occurred at age ≤17 y (n = 6937)^b^
Race/ethnicity		
Non-Hispanic White	1 [Reference]	1 [Reference]
Non-Hispanic Black	2.91 (1.38-6.15)^c^	3.09 (1.36-7.02)^c^
Hispanic: English language survey	3.60 (1.73-7.49)^d^	3.60 (1.59-8.14)^c^
Hispanic: Spanish language survey	1.20 (0.45-3.17)	1.23 (0.44-3.47)
Non-Hispanic Asian or Pacific Islander	0.47 (0.06-3.53)	0.57 (0.08-4.34)
Non-Hispanic multiracial or other race/ethnicities	2.00 (0.63-6.29)	2.37 (0.73-7.67)
Education		
<High school diploma	2.51 (1.18-5.38)^c^	2.99 (1.37-6.53)^c^
≥High school diploma	1 [Reference]	1 [Reference]

Abbreviations: PR, prevalence ratio; SMA, self-managed abortion.

^a^ Sixty-nine participants who refused SMA question were excluded from the analysis.

^b^ Sensitivity analysis to establish that educational attainment preceded SMA attempt. Outcome is ever reported SMA while living in the US, excluding participants who reported using emergency contraception before confirming pregnancy as their only method.

^c^ *P* < .01.

^d^ *P* < .001.

Participants’ mean (SD) age at the time of SMA was 25.4 (6.9) years. The most common methods used for SMA included herbs (38.4% [95% CI, 25.3%-53.4%]), drugs or medications other than misoprostol (29.2% [95% CI, 17.5%-44.5%]), and misoprostol (20.0% [95% CI, 10.9%-33.8%]). A few participants (15.1% [95% CI, 6.9-29.9]) reported using EC after confirming the pregnancy, and 19.8% (95% CI, 10.0%-35.5%) of respondents reported using something physical, such as being hit in the abdomen. Approximately 4 in 10 women (40.8% [95% CI, 27.2%-56.1%]) reported using multiple methods. Less than one-third of respondents (27.8% [95% CI, 16.6%-42.7%]) indicated that the method they used worked to end the pregnancy; 33.6% (95% CI, 21.0%-49.0%) of respondents indicated that they later had a facility-based abortion, 13.4% (95% CI, 7.4%-23.1%) of respondents continued the pregnancy, and 11.4% (95% CI, 4.2%-27.5%) of respondents reported miscarrying later in pregnancy (Table 5). In descriptive analysis, respondents who reported using misoprostol were more likely to indicate that the method worked to end the pregnancy compared with respondents using other drugs, herbs, physical methods, or something else. A total of 11.0% (95% CI, 5.5%-21.0%) of respondents reported experiencing a complication requiring treatment by a physician or nurse; in open-ended responses, 1 respondent reported going to the hospital; however, most respondents who experienced a complication did not give details on the complication (Table 5). Participants reporting a complication used a range of methods, including herbs, drugs or medications, misoprostol, EC before or after confirming the pregnancy, or something else.

**Table 5.** Details of Participants’ Experience Attempting Self-Managed Abortion (SMA)^a^

Characteristic	No. (n = 92)	Weighted % (95% CI)
Age at SMA, y		
≤19	16	15.2 (8.3-26.4)
20-24	21	22.1 (12.3-36.4)
25-29	24	28 (16.8-43)
30-34	12	7.6 (3.5-15.6)
35-39	7	5.9 (2.3-14.4)
≥40	2	1.2 (0.3-5.4)
Missing^b^	10	20.0 (9.4-37.5)
Methods used^c^		
Misoprostol	15	20.0 (10.9-33.8)
EC before confirming pregnancy^d^	21	17.6 (9.5-30.1)
EC after confirming pregnancy	9	15.1 (6.9-29.9)
Other drug or medication	26	29.2 (17.5-44.5)
Herbs^e^	42	38.4 (25.3-53.4)
Physical (eg, hit in abdomen)	15	19.8 (10.0-35.5)
Something else	12	12.6 (6.3-23.6)
Refused	2	2.1 (0.5-8.8)
Decade when SMA took place		
1970s or 80s	5	7.1 (2.4-19.5)
1990s	12	10 (4.7-20.1)
2000s	27	28.6 (17.3-43.5)
2010s	41	44.1 (30.4-58.7)
Missing	7	10.2 (3.6-25.6)
Residence at the time of SMA		
Another country	NA	NA
Same state where currently resides	65	68.5 (53.5-80.5)
Different state than currently resides	23	28.6 (17.0-43.8)
Different but location unclear	4	2.9 (0.9-8.8)
Geographic region of residence at time of SMA		
Northeast (Connecticut, Maine, Massachusetts, New Hampshire, or Rhode Island)	2	1.5 (0.3-7.1)
Mid-Atlantic (New Jersey, New York, or Pennsylvania)	3	3.9 (0.8-17.1)
East N Central (Illinois, Indiana, Michigan, Ohio, or Wisconsin)	14	11 (5.1-22.4)
West N Central (Iowa, Kansas, Minnesota, Missouri, Nebraska, North Dakota, or South Dakota)	5	3.7 (1.1-11.3)
South Atlantic (Delaware, Florida, Georgia, Maryland, North Carolina, South Carolina, Virginia, District of Columbia, or West Virginia)	12	13.4 (5.9-27.7)
East South Central (Alabama, Kentucky, Mississippi, or Tennessee)	3	3.5 (0.8-13.8)
West South Central (Arkansas, Louisiana, Oklahoma, or Texas)	16	21.3 (11.4-36.3)
Mountain (Arizona, Colorado, Idaho, Montana, Nevada, New Mexico, Utah, or Wyoming)	3	2.0 (0.5-8.1)
Pacific (Alaska, California, Hawaii, Oregon, or Washington)	29	36.6 (23.7-51.8)
Refused	5	3.1 (1.0-8.8)
Effectiveness of SMA in ending pregnancy		
Successful	27	27.8 (16.6-42.7)
Unsuccessful, had abortion at clinic or hospital	29	33.6 (21.0-49.0)
Unsuccessful, continued pregnancy	18	13.4 (7.4-23.1)
Unsuccessful, subsequently had miscarriage	5	11.4 (4.2-27.5)
Undetermined	12	13.3 (6.8-24.7)
Refused	1	0.5 (0-3.4)
Experienced any complications requiring treatment		
Yes	12	11.0 (5.5-21.0)
No	80	89.0 (79.0-94.5)
Refused	0	NA
Reasons for SMA^c^		
Did not know where a clinic was	10	8.4 (3.3-19.6)
Clinic too expensive	30	25.2 (15.7-37.7)
Clinic too far away	15	13.0 (7.1-22.8)
Doing on own seemed easier or faster	37	47.2 (33.0-61.8)
Thought needed parent’s consent	10	13.7 (6.8-25.8)
Doing on own seemed natural	13	13.2 (6.2-26.2)
Use vitamins or herbs whenever sick	11	6.4 (2.8-13.9)
Other	16	16.7 (8.4-30.9)

Abbreviations: EC, emergency contraception; NA, not applicable; SMA, self-managed abortion.

^a^ Analysis limited to 92 participants who reported attempting SMA while living in the US and using a method other than EC before confirming their pregnancy.

^b^ Missing data on 7 participants who refused timing question and 3 participants who provided an illogical response based on current age.

^c^ Proportions do not sum to 100 because women could select multiple response options and write in responses.

^d^ These participants also reported using another method along with EC before confirming pregnancy.

^e^ The herbs used including parsley (8 respondents), cohosh (5 respondents), vitamin C (5 respondents), tea/chamomile (4 respondents), and cinnamon, pennyroyal, dong quai, papaya, and oregano (2-3 respondents each). Others indicated that they did not remember (9 respondents) or did not know (2 respondents) what they used.

The most common reasons reported for attempting SMA were that it seemed easier or faster (47.2% [95% CI, 33.0%-61.8%]) or that facility-based care was too expensive (25.2% [95% CI, 15.7%-37.7%]). Respondents less frequently cited concerns about needing a parent’s consent (13.7% [95% CI, 6.8%-25.8%]) and the clinic being too far away (13.0% [95% CI, 7.1%-22.8%]). Some respondents cited SMA as feeling more natural (13.2% [95% CI, 6.2%-26.2%]) (Table 5).

## Discussion

To our knowledge, this cross-sectional study presents the first national, population-based estimate of experience with SMA in the US. We found that 1.4% (95% CI, 1.0%-1.8%) of self-identified women ages 18 to 49 years reported having ever tried to end a pregnancy on their own outside of the health care system. Adjusting for estimated underreporting of abortion, that figure is 2.0%. Based on 2017 Census population estimates,^22^ this indicates that approximately 900 000 to 1.3 million US women of reproductive age have experience with SMA. Assuming current abortion rates stay constant and that SMA attempts are underreported at the same rate as abortion in general, we project that 7.0% (95% CI, 5.6%-8.4%) of US women will attempt SMA in their lifetime.

Prior research on SMA in the US has frequently focused on the experiences of Latinx individuals, in particular those living near the border with Mexico.^3,4,17^ Consistent with this research, we find higher levels of SMA experience among those who identify as Hispanic, particularly those who took the survey in English, and among foreign-born individuals overall.^14^ A novel contribution of this study is its use of a nationally representative sample, which allows for further exploration into who attempts SMA. Notably, the prevalence of SMA among Black women was nearly 3-fold times greater than among Non-Hispanic White women. Although our study does not elucidate the reasons underlying these patterns, we hypothesize that the same systems that prevent Black and Hispanic individuals from accessing other forms of reproductive health care and that may contribute to disparities in unintended pregnancy rates,^29^ including structural racism, discrimination, stigma, and cost,^30,31,32,33,34^ might also prevent them from accessing a facility-based abortion or foster a preference for SMA outside the health care system.

Approximately 1 in 5 participants indicated that the reason they attempted to SMA was because they could not locate a clinic or would have to travel too far to get to a clinic. Abortion deserts—regions of the US where people have to travel more than 100 miles to an abortion clinic—are already common, particularly in the Southern and Midwestern US^35^ Several factors, including unprecedented levels of new state-level abortion restrictions,^36^ the Trump administration’s domestic gag rule restricting primary and reproductive health care clinicians’ ability to discuss or refer patients to abortion,^37,38^ and the potential for a Supreme Court decision that could reshape federal Constitutional protections on abortion,^39^ suggest an urgent need to pay close attention to trends in SMA, both overall and by region, in the coming years.

One-third of participants who had ever attempted SMA indicated that their attempt was unsuccessful and they sought a facility-based abortion. This suggests that SMA attempts identified at the time of abortion care–seeking may represent only one-third of all SMA attempts; accordingly, estimates of SMA among people recruited in clinic settings, which currently range from 2% to 13% depending on the study,^4,5,15^ should be interpreted as underestimates of the frequency of SMA.

Finally, our results highlight how SMA encompasses many different types of methods with vastly different and sometimes unknown^40^ levels of safety and effectiveness. Consistent with prior research,^4^ herbs were the most common method used. However, others took misoprostol, drugs or medications other than misoprostol, EC after confirming a pregnancy, or used a physical method, often in combination. Sample sizes were too small to evaluate whether there were statistically significant differences in safety and effectiveness by method used. However, notably, complications were not common, despite use of some methods with the potential for harm.

### Limitations

There were limitations to this study. Abortion is a stigmatized and underreported health behavior.^26^ We compare past-year reports of abortion to national data from clinics in an attempt to adjust for underreporting; this method assumes that people underreport their SMAs at a similar rate as facility-based abortions. However, we cannot empirically test this assumption, leaving open the possibility that our results adjusted for underreporting are inaccurate. Approximately one-half of GfK panel members invited to complete our survey participated, and there was evidence of underrepresentation of people living in poverty in our sample compared with the NSFG. However, this suggests that we are underestimating SMA prevalence, as SMA experience was more common among people living at less than 100% of FPL. Our question assessing the methods used to SMA did not specifically list mifepristone as a response option. As a result, participants may have described use of mifepristone in the “other drug” category. Furthermore, we excluded use of EC prior to pregnancy confirmation as a method of SMA because this may have represented appropriate use of the method; however, if EC was not used as indicated, we may again be underestimating SMA. Response rates for the open-ended prompt on complications were low; future research should consider use of a validated measure of complications or adverse events.^41^ Despite cognitive work to ensure a comprehensive definition of SMA, it remains possible that some people reported medication abortion obtained from a clinician as self-managed care. Additionally, we did not ask participants whether and how they confirmed their pregnancy, so it is possible that some respondents are describing methods used while experiencing a pregnancy scare. This is not a limitation for developing accurate estimates of experience attempting SMA, but it does introduce caution into interpreting our estimates of method effectiveness.

## Conclusions

There has been much attention focused on the continued decline in the US abortion rate, but that estimate only counts abortions occurring in facilities.^12^ This national cross-sectional survey study provides further evidence that SMA is occurring outside of the formal health care system, and people of color, those with lower incomes, and those who face barriers to care were more likely to attempt SMA. As abortion clinics close owing to increased abortion restrictions, a reduced demand for facility-based abortions,^39^ and a growing demand for convenience, privacy, and the comfort of self-managed abortion^9^ and self-care more broadly,^42^ it is likely that SMA will become more prevalent in the US, as it is today in other countries.^13^ This national estimate serves as an important baseline to track this phenomenon moving forward.

## Notes

Supplement. **eFigure 1.** Flowchart of Inclusion in Primary Outcome of Lifetime Attempt to Self-Manage Abortion (SMA) **eTable.** Sociodemographic Profiles of the National Survey of Family Growth, 2015 to 2017, and the GfK KnowledgePanel populations **eFigure 2.** Age-Specific Rates of Self-Managed Abortion, Projected Using Discrete-Time Event Models
