# Supplementary material for: Prevalence of Self-Managed Abortion Among Women of Reproductive Age in the United States
Source: JAMA Netw Open. 2020 Dec 18;3(12):e2029245. doi: 10.1001/jamanetworkopen.2020.29245 (PMC7749440; doi:10.1001/jamanetworkopen.2020.29245)
Supplement: Supplement. — eFigure 1. Flowchart of Inclusion in Primary Outcome of Lifetime Attempt to Self-Manage Abortion (SMA) eTable. Sociodemographic Profiles of the National Survey of Family Growth, 2015 to 2017, and the GfK KnowledgePanel populations eFigure 2. Age-Specific Rates of Self-Managed Abortion, Projected Using Discrete-Time Event Models [file jamanetwopen-e2029245-s001.pdf]

Supplemental Online Content

Ralph L, Foster DG, Raifman S, et al. Prevalence of self-managed abortion among women of reproductive age in the United States. *JAMA Netw Open*. 2020;3(12):e2029245. doi:10.1001/jamanetworkopen.2020.29245

eFigure 1. Flowchart of Inclusion in Primary Outcome of Lifetime Attempt to Self-Manage Abortion (SMA)

eTable. Sociodemographic Profiles of the National Survey of Family Growth, 2015 to 2017, and the GfK KnowledgePanel populations

eFigure 2. Age-Specific Rates of Self-Managed Abortion, Projected Using Discrete-Time Event Models

This supplemental material has been provided by the authors to give readers additional information about their work.

© 2020 Ralph L et al. *JAMA Network Open.*

eFigure 1. Flowchart of Inclusion in Primary Outcome of Lifetime Attempt to Self-Manage Abortion (SMA)

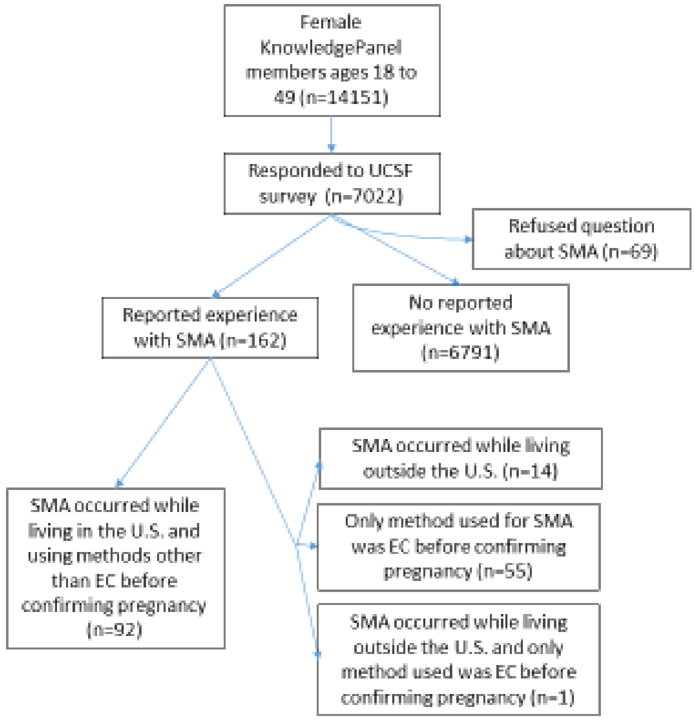

Notes: SMA= self-managed abortion, EC=emergency contraception

© 2020 Ralph L et al. *JAMA Network Open.*

eTable. Sociodemographic Profiles of the National Survey of Family Growth, 2015 to 2017, and the GfK KnowledgePanel populations

				National Survey of Family Growth, 2015-17 (N=5012)	GfK Sample, 2017 (N=7022)
				Weighted %	Weighted %
Percent of Federal Poverty Level					
	<100%			22.0	15.2
	100 to 199%			22.4	16.0
	≥ 200%			55.5	68.8
Age (years)					
	18-19			6.2	4.6
	20-24			15.2	14.8
	25-29			16.7	16.8
	30-34			16.0	16.5
	35-39			15.5	15.8
	40-44			14.7	15.2
	45-49			15.7	16.2
Race/ethnicity					
	Non-Hispanic white			58.8	57.1
	Non-Hispanic Black			14.5	13.1
	Non-Hispanic other			6.6	9.5
	Hispanic			20.1	20.3
Language of the survey					
	English			89.7	91.1
	Spanish			7.9	8.9
	Other[a]			2.4	--
Highest level of education completed					
	< High school (HS)			10.3	10.0
	HS diploma or GED			24.6	22.8
	Some college or associate's degree			23.6	32.0
	College degree			41.5	35.2
Marital status					
	Married			43.4	50.4
	Widowed/Divorced/Separated			11.3	6.6
	Never married			31.2	31.8
	Living with partner			14.2	11.2

© 2020 Ralph L et al. *JAMA Network Open.*

Parity					
	0			37.4	45.5
	1			17.8	16.0
	≥ 2			44.9	38.5
Notes: The NSFG sample is restricted to participants aged 18 to 49; Weighted percents represent proportions obtained after applying survey weights provided by the NSFG and GfK; [a]The GfK survey is only administered in English and Spanish.					

© 2020 Ralph L et al. *JAMA Network Open.*

eFigure 2. Age-Specific Rates of Self-Managed Abortion, Projected Using Discrete-Time Event Models

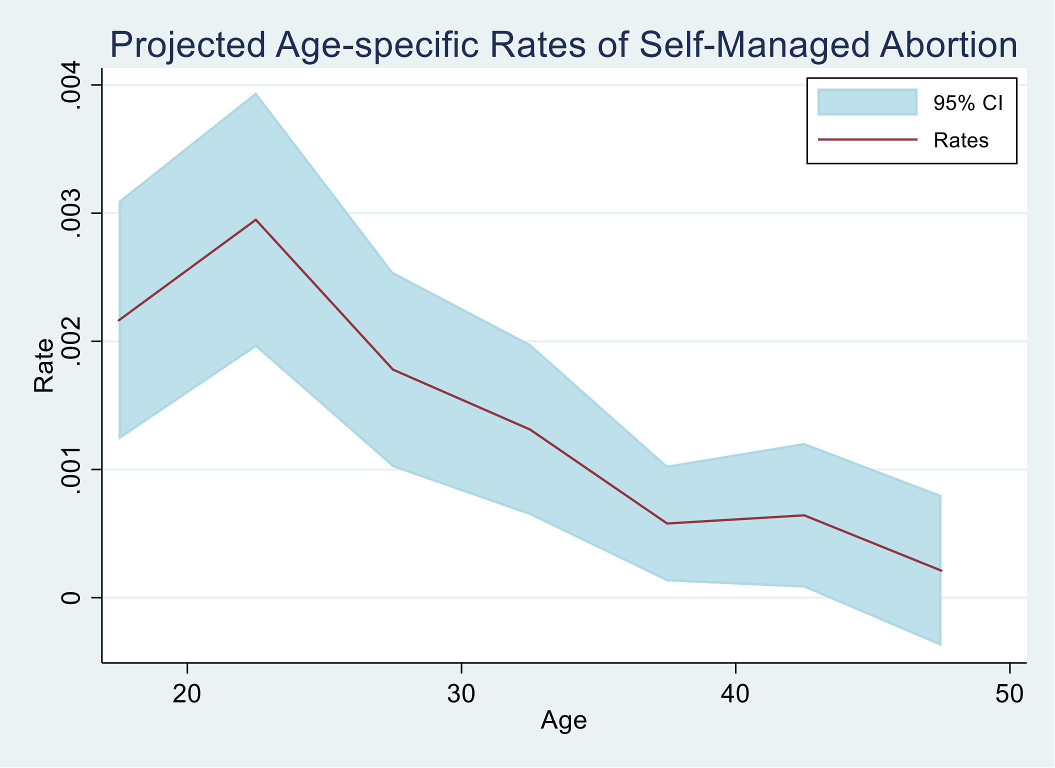

© 2020 Ralph L et al. *JAMA Network Open.*
